# Grouped Beating in Eisenmenger: Successful Localization and Ablation of an Accelerated Idioventricular Rhythm from Within the Purkinje System

**DOI:** 10.1016/s0972-6292(16)30631-3

**Published:** 2013-06-25

**Authors:** Shohreh Honarbakhsh, Irina Suman-Horduna, Lilian Mantziari, Sabine Ernst

**Affiliations:** 1 Royal Brompton and Harefield NHS Foundation Trust and Imperial College London, United Kingdom; 2 NIHR Cardiovascular Biomedical Research Unit, Royal Brompton and National Heart and Lung Institute, Imperial College London (SE)

**Keywords:** Bundle Branch Block, Linking

## Abstract

A 33-year old female with a background of Eisenmenger syndrome secondary to multiple congenital muscular ventricular septal defects (VSD) was admitted with a recent history of frequent intermittent palpitations. It was noted that she had an independent accelerated idioventricular rhythm (AIVR), with rates varying between 85-110bpm, which exhibited a repetitive grouped beating pattern. Although generally perceived as benign, in this case this rhythm was drug refractory, was associated with significant compromise to cardiac filling and output and progressed to haemodynamically intolerable sustained ventricular tachyarrhythmia. Successful ablation was performed at the inferior aspect of the residual VSD, from within the Purkinje network.

## Case Presentations

A 33-year-old female with a background of Eisenmenger syndrome secondary to multiple congenital muscular ventricular septal defects (VSDs) was admitted with a one-day history of frequent intermittent palpitations. There were no precipitating factors. She was diagnosed with VSDs during childhood and initially underwent pulmonary artery banding followed by surgical VSD repair and pulmonary debanding. However she had residual VSD patency and developed post-operative complete heart block requiring dual chamber pacemaker implantation. She had a previous history of right-sided atrial tachycardia treated with external cardioversion. Her most recent pacemaker interrogation showed atrial flutter and the patient initially underwent a successful cavo-tricuspid isthmus ablation.

**Figure 1:**
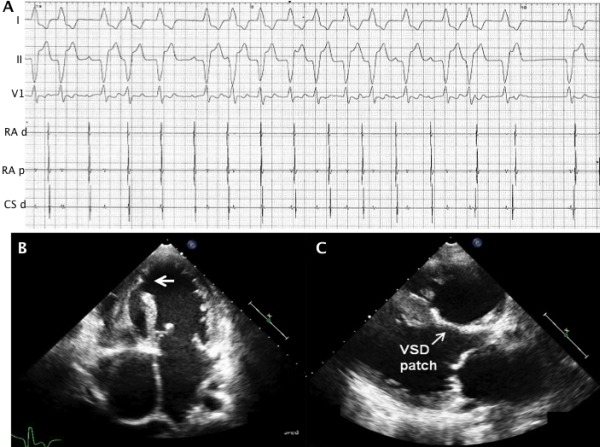
A Tracing recorded at the end of the first ablation procedure for cavotricuspid isthmus-dependent atrial flutter, showing an accelerated idioventricular rhythm with variable cycle length along with corresponding endocavitary signals. Note the presence of ventriculo-atrial dissociation. Paper speed is 25mm/sec. RA d, RA p - right atrial distal and proximal; CS d - coronary sinus distal. B and C illustrate transthoracic apical 4 chamber (B) and parasternal long axis (C) echocardiographic views. The apical residual ventricular septal defect is arrowed and measures 1.1cm.

At the time of the procedure and post-procedure it was noted that she had an independent accelerated idioventricular rhythm (AIVR), with rates varying between 85-110 bpm (Figure 1A) with occasional transient acceleration up to 150 bpm. This AIVR was initially not associated with any symptoms. The cycle length of the AIVR was variable, but exhibited a repetitive grouped beating pattern with occasional progressively shorter RR intervals followed by longer cycles. It had a LBBB morphology suggesting that it had a predominant right sided origin. This rhythm was dissociated from a slower sinus activity and its origin was mapped close to the inferior border of the residual VSD. Attempted ablation of this rhythm was not made initially and medical management was first considered.

Transthoracic echocardiogram post atrial flutter ablation procedure showed moderately dilated right ventricle with moderately impaired function, severe tricuspid regurgitation, a mean pulmonary artery pressure of 52 mmHg, an apical patent VSD (Figure 1B, arrowed) and no residual flow across the previously closed VSD (Figure 1C). The left ventricle showed moderate systolic dysfunction with ejection fraction of 43%. Notably, a significantly impaired filling due to reduced atrial emptying during ventricular systole and compromised cardiac output were demonstrated in the presence of frequent ventricular arrhythmia. As there was no significant change to the AIVR with up-titration of beta-blocker and increased atrio-ventricular sequential pacing rate to 90 bpm, along with it being associated with reduced cardiac function, an elective ablation was scheduled. Prior to this however, after having been almost continuously in this rhythm for 5 days without significant associated symptoms, the patient developed sustained ventricular tachycardia (VT) with the same morphology as the AIVR and a rate up to 188 beats/min with haemodynamic compromise, which was terminated with intravenous lignocaine. She remained in the AIVR following the intravenous lignocaine and subsequently underwent an emergency ablation procedure.

## Invasive electrophysiological study and ablation

At the beginning of the study the patient had monomorphic ventricular ectopies and repetitive runs of idioventricular rhythm with grouped beating behavior. The rhythm could not be terminated by overdrive, resumed at a similar rate after ventricular overdrive pacing and showed spontaneous acceleration/deceleration, suggestive of enhanced automaticity.

**Figure 2:**
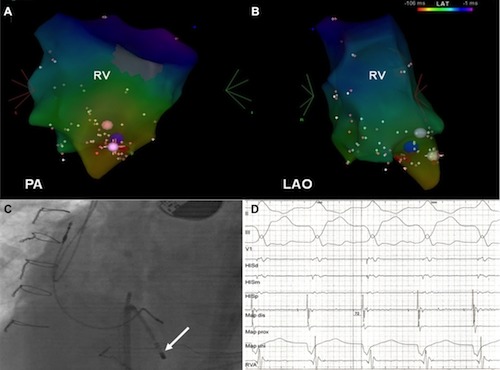
A and 2B display postero-anterior (C) and left anterior oblique (D) views of the acquired 3D activation map of the right ventricle during the idioventricular rhythm. It illustrates the centrifugal spread of activation from an apico-septal focus. The successful ablation site is marked by a blue dot. C illustrates the left anterior oblique catheter position of the mapping catheter (arrowed) apico-septally, at the border of the ventricular septal defect. D. Bipolar and unipolar map recordings are displayed at the successful ablation site during the idioventricular rhythm. The distal bipole of the ablation catheter (Map d) records a small sharp potential, preceding the QRS onset by 72ms. Sweep speed is 100mm/sec. His d - His distal; His m - His middle; His p - His proximal; Map d - map distal; Map p - map proximal; Map uni - map unipolar; RVA - right ventricular apex.

Mapping of the right ventricle was performed via a steerable sheath (Agilis, SJM) on the CARTO 3D electroanatomical mapping system (Biosense Webster, Brussel) and activation mapping performed during the idioventricular rhythm depicted the earliest ventricular activation apico-septally at the inferior aspect of the residual VSD (Figure 2A, 2B and 2C), with a radial spread of activation. At the earliest site, a consistent small sharp potential was recorded that preceded the onset of the QRS complex by 72 ms (Figure 2D). At this site, located at the inferior margin of the VSD, irrigated-tip ablation (30 ml/min, 40 watts, 2 minutes) resulted in immediate termination of the arrhythmia (within 5 seconds) into sequential atrio-ventricular paced rhythm. There was no further recurrence during the 90 min waiting time with and without Isoprenaline infusion.

The patient remained symptom free during continuous monitoring for 72 hours and she was scheduled for an upgrade to an implantable defibrillator for secondary prevention.

## Discussion

We report a case of a 33 year old female with Eisenmenger syndrome presenting with a persistent drug refractory AIVR that resulted in a significant compromise to cardiac filling and output and which progressed to haemodynamically intolerable sustained VT. With the use of the 3D electoanatomical mapping we were able to successfully localize its focus anatomically at the inferior border of the VSD and demonstrate its origin within the distal electrical conduction system.

AIVR is related to enhanced automaticity in His-Purkinje fibers or ventricular myocardial cells [1]. It is predominantly transient in nature, usually causes no haemodynamical compromise and seen as a benign transient arrhythmia (2). AIVRs have been reported in patients during the reperfusion phase following acute myocardial event (3), can be secondary to certain drugs such as digitalis toxicity [2] and be associated with congenital heart disease [4] but has also been seen occasionally post-surgery or discovered fortuitously in asymptomatic patients with apparently normal hearts.

In this case, the morphology of the AIVR was LBBB suggestive that it had a predominant right sided origin. Existing literature predominantly focuses on purkinje activity originating from the left ventricle. Additionally in this case, this rhythm occurred without any obvious precipitating factors in a post-surgical patient with Eisenmenger physiology and originated close to the margins of the VSD. The peculiar behavior of repetitive grouped beating pattern suggested an enhanced automaticity in the electrical conduction system with intermittent conduction block towards the ventricular myocardium, similar to a Wenckebach phenomenon [5], which resulted in progressively shorter RR intervals until the conduction block occurred (longer RR intervals). Indeed, the electrophysiological study confirmed the origin of the arrhythmia from within the His-Purkinje system and we effectively completely ablated its focus by radiofrequency current.

It has been reported that the rate of the idioventricular rhythm was related to the probability of developing life threatening arrhythmias and increased mortality [2]. Pre-existent biventricular dysfunction, raised right ventricular systolic and diastolic pressures or decreased coronary flow secondary to decreased cardiac output caused by the AIVR may account for some of the possible mechanisms which precipitated acceleration and degeneration of this rhythm into a faster VT in this particular patient with fragile Eisenmenger physiology. In such drug refractory cases early aggressive control including radiofrequency ablation should be sought, especially if this rhythm is associated with signs of compromised cardiac output.
